# A Comparative Analysis of Mechanical Properties in Injection Moulding (IM), Fused Filament Fabrication (FFF), and Arburg Plastic Freeforming (APF) Processes

**DOI:** 10.3390/polym17070990

**Published:** 2025-04-05

**Authors:** Caolan Jameson, Declan M. Devine, Gavin Keane, Noel M. Gately

**Affiliations:** 1 PRISM Research Institute, Technological University of the Shannon: Midlands Midwest, Athlone Campus, University Road, N37 HD68 Athlone, Ireland; caolan.jameson@tus.ie (C.J.); declan.devine@tus.ie (D.M.D.); 2 Centre for Industrial Service & Design, Technological University of the Shannon: Midlands Midwest, Athlone Campus, University Road, N37 HD68 Athlone, Ireland; gavin.keane@tus.ie

**Keywords:** injection moulding, Arburg plastic freeforming (APF), fused filament fabrication (FFF), polycarbonate/acrylonitrile butadiene styrene (PC/ABS), additive manufacturing, mechanical properties

## Abstract

This study explores the mechanical performance of polycarbonate (PC) and acrylonitrile butadiene styrene (ABS) filaments fabricated using fused filament fabrication (FFF), Arburg plastic freeforming (APF), and injection moulding (IM). A series of controlled experiments, including differential scanning calorimetry (DSC), scanning electron microscopy (SEM), dynamic mechanical thermal analysis (DMA), and mechanical tests, were conducted to evaluate the material’s mechanical, thermal, and chemical properties. The results highlight the influence of process parameters and material choice on the mechanical properties of PC/ABS components. The FFF samples exhibited the highest impact strength (up to 28.82 kJ/m²), attributed to porosity acting as a stress absorber under impact load. However, this same porosity led to a 9.14% and 19.27% reduction in flexural and tensile strength, respectively, compared to the APF samples, where stress concentration effects were more pronounced under flexural loads. APF’s mechanical properties were comparable to those of IM, with the process achieving the highest tensile strength, highlighting its potential for producing robust PC/ABS samples. This study aims to provide valuable insight into the selection of additive manufacturing (AM) processes for PC/ABS components.

## 1. Introduction

Additive manufacturing (AM), commonly known as 3D printing, comprises a wide range of processing techniques which allow for custom components to be made with complex geometries. Traditional approaches to polymer manufacturing such as injection moulding (IM) require custom-made moulds, which have a high initial cost and typically have long lead times. These factors are critical when producing low volume parts or rapid prototyping, which makes AM an ideal process. In terms of polymer-based 3D printing, fused filament fabrication (FFF) is one of the most common AM processes, and the mechanical properties of these manufactured components have been studied in detail [1,2]. Processing parameters such as layer thickness [3], infill pattern, infill percentage [4], print speed, build orientation, and nozzle temperature have a significant effect on the mechanical properties of FFF components. The FFF process uses filament feedstock materials, which limits the range of usable materials [5]. Arburg plastic freeforming (APF) is an AM technique that uses droplet deposition modelling to form 3D-printed components layer-by-layer [6,7]. While AM processes such as FFF and APF can create custom parts, the APF process allows for a wider range of material selection due to a plasticizing screw similar to that of an IM machine. The most common material studied in APF is acrylonitrile butadiene styrene (ABS) [5,8,9,10,11,12,13] with various materials such as poly(lactic acid) (PLA) [14], polycarbonate (PC) [15], thermoplastic polyurethane (TPU) [6], poly(methyl methacrylate) (PMMA) [16,17], poly(ethylene oxide) (PEO)/polyvinylpyrrolidone-vinyl acetate (PVPVA)/polycaprolactone (PCL) [18,19], poly(lactic-co-glycolic acid) (PLGA) [20], polypropylene (PP) [21], and PC/ABS [22] also being studied.

Among the various polymer-based 3D printing techniques, there are many examples of comparisons between techniques in the literature. Bassand et al. [20] compared the APF and FFF processes as suitable manufacturing techniques for PLGA implants. While finding that both can be used to produce implants with drug-releasing capabilities, the authors reported that the deposited droplets in the APF samples led to differences in the porosity of the mesh and drug release kinetics. Minetola et al. [13] compared APF to traditional AM techniques such as FFF and SLS in terms of dimensional accuracy and found that APF performed better for larger features when compared to FFF printing. However, for this comparison, each AM technique used varied materials, with PA 12, CO-polyester, and ABS being used in SLS, FFF, and APF processes, respectively. The dimensional stability of these polymers can vary, which could have affected the dimensions of the features being tested. Hecker et al. [10] compared the geometric and mechanical properties and economic efficiency of ABS samples manufactured using the FFF and APF processes. They found that the mechanical properties of the APF samples were higher than those of the FFF process except in the Z orientation, where tensile strength was higher for the FFF samples. This comparison used an ABS-M30 filament and a layer height of 0.178 mm. This layer height was preselected for the Fortus 400 mc, with APF having a stock layer height of 0.2 mm. This reduction in layer height without changing the DAR values could have affected the part density, resulting in lower properties. Pinter et al. [9] compared the APF, FFF, and IM processes with ABS (Terluran GP 35) and found that the APF samples had a more consistent density compared to the FFF printed samples, which led to more consistent mechanical properties. This inconsistency in density in FFF samples was determined to be because of the variation in filament diameter.

However, to the best of our knowledge, there is no published literature on the comparison of the mechanical properties of PC/ABS samples manufactured using FFF, APF, and IM processes. This study aims to qualify a PC/ABS filament on the Arburg freeformer and compare the mechanical properties to those of IM and fused filament fabrication.

## 2. Materials and Methods

### 2.1. Materials

A blend of polycarbonate (PC) and acrylonitrile butadiene styrene (ABS) was supplied in 2.85 mm filament form by RS components (RS group, Corby, United Kingdom). The density of the PC/ABS filament was 1.05 g/cm^3^, with a melting temperature range of 240–260 °C and a print temperature of 270 °C.

For tensile, impact, and flexural testing, samples were manufactured in accordance with ASTM D638-14 type I [23], D6110-10 [24], and D790-10 [25] specifications, respectively. These samples were printed individually in the XY and XZ orientations.

### 2.2. Material Processing

#### 2.2.1. Fused Filament Fabrication (FFF) Printing

Samples were manufactured using the BCN3D Sigmax R19 FFF printer (BCN3D Technologies Inc., Barcelona, Spain). The following printing parameters were utilized for all the samples printed: nozzle temperature of 270 °C, build plate temperature of 100 °C, layer height of 0.2 mm, printing speed of 60 mm/s, infill density of 100%, line infill pattern, and brim build plate adhesion.

#### 2.2.2. Injection Moulding (IM)

The PC/ABS filament was pelletized and dried at 80 °C for 4 h prior to IM. IM was performed using the Arburg Allrounder IM machine (Arburg GmbH + Co KG, Lossburg Germany). The temperature profile was set at (from die to feeder) 265/260/240/240/230/50 °C. The following moulding parameters were utilized for all samples: mould temperature of 75 °C, back pressure of 40 bar, injection pressure of 1500 bar, injection speed of 40 mm/s, holding pressure of 1100 bar, holding time of 5 s, and cooling time of 30 s.

#### 2.2.3. Arburg Plastic Freeforming (APF)

Sample manufacturing was performed on an Arburg freeformer (APF) 300-3X (Arburg GmbH + Co KG, Lossburg, Germany) additive manufacturing machine. The PC/ABS filament was pelletized and dried at 80 °C for 4 h. A 0.2 mm diameter nozzle was used. The temperature profile for APF was set at (from nozzle to feeder) 250, 230, 220, 45 °C with a build chamber temperature of 100 °C. A starting droplet aspect ratio (DAR) range was determined by measuring droplet height and width in the absence of any external force on the droplet. An optical microscope was used to determine the DAR range. An experimental DAR was obtained by manufacturing 20 mm cubes with varying DAR values, from 1.1 to 1.26, to test for print quality and density. Once the DAR was determined, the following process parameters were utilized for all samples: discharge rate of 50%, Z offset of 1.4 mm, layer thickness of 0.2 mm, and DAR of 1.1.

### 2.3. Archimedes’ Buoyancy Method

The density of the manufactured samples was determined using ASTM D792 standards *n* = 5. First, the weight of the samples in air was measured, and the sample was submerged in water. The weight of the sample in water was measured, and after applying the correction factors for the density of air and water at 20 °C, the density of the manufactured samples was determined. For the APF samples with varying DAR values, the experimentally determined density was compared to the theoretical density supplied by the manufacturer. A minimum of 5 samples were measured for each process/orientation.

### 2.4. Scanning Electron Microscopy (SEM)

Scanning electron microscopy was conducted using a Mira XMU SEM (Tescan Brno, Czech Republic) in backscattered electron mode to determine the shape and distribution of the internal porosity of the manufactured parts. The accelerated voltage was set at 20 kV for all materials. Before analysis, the test samples were sputtered with gold using a Baltec SCD 005 for 110 s at a 0.1 mbar vacuum.

### 2.5. Differential Scanning Calorimetry (DSC)

A NETZSCH DSC 214 Polyma (Erich NETZSCH B.V. and Co. Holding KG, Selb, Germany) was used to detect the glass transition temperature (Tg) of virgin filament and for each process/orientation. The samples were tested in duplicate at a heating rate of 5 °C/min, from ambient to 300 °C. The samples were held at 300 °C for 10 min to remove thermal history and cooled to ambient at a rate of 5 °C /min. A second heating cycle was also assessed at a rate of 5 °C /min to 300 °C. Aluminium standard NETZCH pans and lids and a sample weight of 5–10 mg were used.

### 2.6. Dynamic Mechanical Thermal Analysis (DMA)

Dynamic mechanical thermal analysis was used to measure the storage modulus, loss modulus, and loss factor (tan delta), and these were determined using a TA Instruments Q800 DMA (TA Instruments, Eschborn, Germany) and a dual cantilever apparatus. The analysis was done at a frequency of 1 Hz. The temperature range was ambient temperature to 150 °C at a heating rate of 3 °C/min. A minimum of 5 samples were tested for each process/orientation.

### 2.7. Melt Flow Index (MFI)

The melt flow index of the PCABS filament was measured using a Zwick Rowell Cflow extrusion plastometer (ZwickRoell Ltd., Ulm, Germany). The temperature was set at 270 °C, and, using ISO 1133-1:2022 standards [26], a standard weight of 5 kg was used for the test. A die with a nominal length of 8 mm and a bore diameter of 2.095 mm was used. Samples were cut every 30 s, with the weight of the samples measured using a laboratory scale. Five replicates were taken for each process/orientation.

### 2.8. Charpy Impact Testing

Charpy impact tests were conducted using the Ceast Resil 5.5 impact tester (Illinois Tool Works Inc., Glenview, IL, USA). The impact properties were determined in accordance with the ASTM D6110-10 standards [24]. A 4-joule hammer was used. A minimum of 10 samples were tested for each process/orientation.

### 2.9. Tensile Testing

The samples were tested using the Zwick Roell Z010 with a load cell of 10 kN (ZwickRoell Ltd., Ulm, Germany). The tests were carried out at room temperature. The tensile properties were determined in accordance with the ASTM D638-14 standards [23]. The test speed was set to 5 mm/min, and the gauge length was 50 mm. A minimum of 10 samples were tested for each process/orientation. A video extensometer was used to measure the modulus of the materials.

### 2.10. Flexural Testing

Flexural tests were conducted using the Zwick/Roell Z010 (ZwickRoell Ltd., Ulm, Germany). The flexural properties were determined in accordance with the D790-10 standards [25]. A span-to-depth ratio of 16:1 and a test speed of 2.7 mm/min were utilized. A minimum of 10 samples were tested for each process/orientation.

### 2.11. Statistical Analysis

The statistical analysis of the density, tensile, impact, and flexural measurements was carried out using 2-way analysis of variance (ANOVA) in Minitab 20.2 statistical software (Minitab Ltd., Coventry, UK). All the values were evaluated using a 95% confidence interval with a significance level of *p* ≤ 0.05.

## 3. Results

### 3.1. DAR vs. Density Assessment

The relationship between DAR and part density was assessed using the Archimedes principle of the density of solids when submerged in a known liquid. The aim was to define a DAR value that represents the known density of the material of 1.05 g/cm^3^, which will achieve a 100% filled part. The DAR is the ratio of the width (W) to the height (H) of the droplet (DAR = W/H). The DAR is one of the most important variables for the APF process [17]. It can be affected by processing conditions such as nozzle temperature, which reduces the viscosity of the material [16]. A lower nozzle temperature of 250 °C was set on the APF nozzle compared to the FFF printing nozzle of 270 °C due to the increase in the flow rate of the material at the FFF printing temperature through the APF nozzle. At the FFF printing temperature, no droplets formed as the material came through the nozzle as a continuous filament. The nozzle temperature was reduced by 20 °C, at which point droplet formation was observed using an optical microscope.

**Figure 1:**
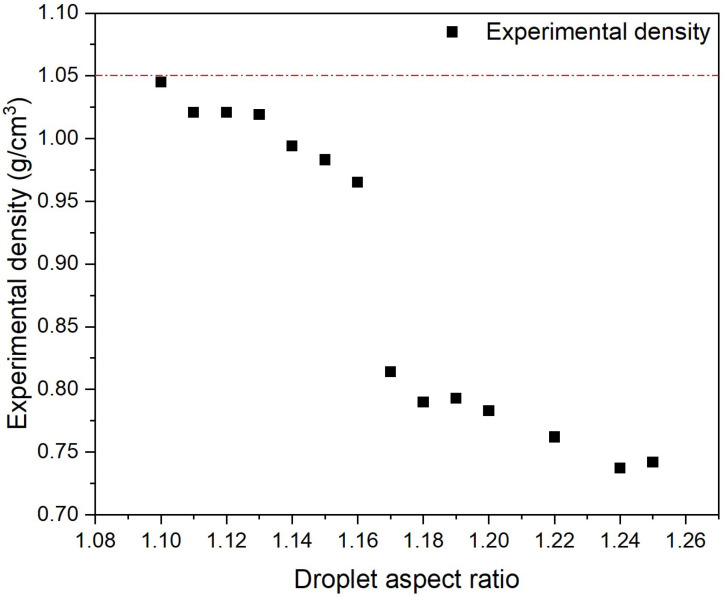
Droplet aspect ratio vs. density of APF-produced parts.

Samples were printed as cubes of 20 × 20 × 20 mm with varying DAR values from 1.1 to 1.25, and the density of each sample was assessed (Figure 1). For this PC/ABS filament, the closest value to achieving 100% density was a DAR value of 1.1, which achieved a density of 1.045 g/cm^3^. This value represents 99.52% of the known density of the material provided by the supplier. Samples with a DAR lower than 1.1 kept overpacking the part, resulting in printing failure caused by the discharge rate being below tolerance. The DAR value of 1.1 was chosen, and samples printed using the material in this study used this value.

**Figure 2:**
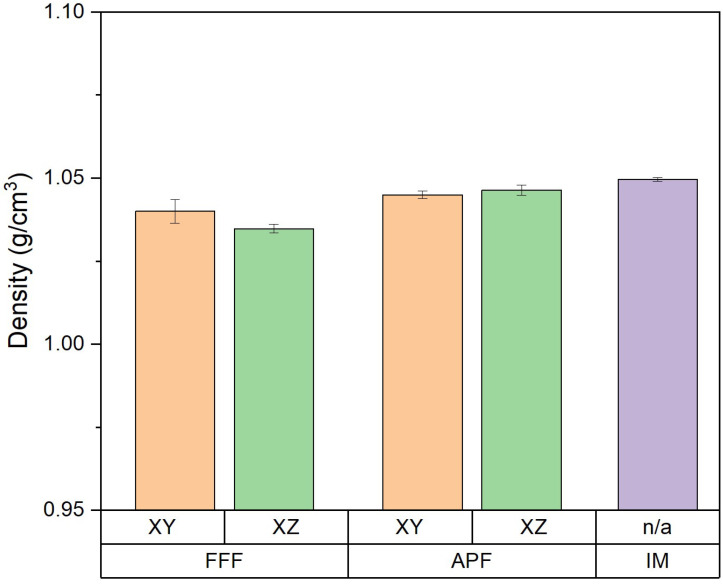
Comparison of density for the three processes in both orientations (*n* = 5).

Samples manufactured using APF were assessed in both the XZ orientation ($\rho =1.0464\;g{/cm}^{3}$) and the XY orientation ($\rho =1.0450\;g{/cm}^{3}$), with no significant difference being recorded (*p* = 1.000). The density of the impact samples manufactured using IM and FFF was also assessed using Archimedes’ principle (Figure 2). There was a significant difference observed between the FFF samples and any other sample produced. For the FFF samples in the XY orientation ($\rho =1.04\;g{/cm}^{3}$) and the XZ orientation ($\rho =1.0348\;g{/cm}^{3}$), we observed a significant difference (*p* = 0.003). Samples manufactured using APF in the XZ orientation and using IM ($\rho =1.0496\;g{/cm}^{3}$) had no significant difference recorded (*p* = 0.168).

### 3.2. Scanning Electron Microscopy

**Figure 3:**
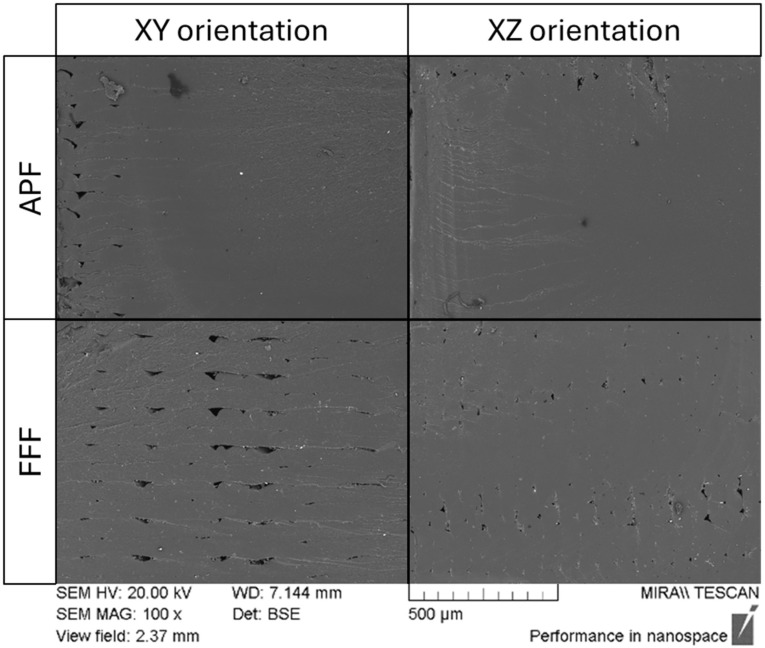
SEM images of the APF and FFF samples in the XY and XZ orientations at 100× magnification.

The porosity of the samples manufactured using APF and FFF was also assessed using scanning electron microscopy (SEM) (Figure 3) along a single fracture line of an impact sample. While the samples manufactured using APF in the XY orientation and the XZ orientation show a visual difference in void content, there was not a significant difference in density. A significantly higher density for samples manufactured using FFF in the XY orientation compared to the XZ orientation was observed, with a higher concentration of voids along each layer (*p* = 0.003). The density and SEM results suggest that printing in the XZ orientation would reduce the density for FFF-printed samples, as an increase in the number of layers would increase the chance of inter-layer voids and the number of voids at the contours. The location of the voids differs for each process. Samples manufactured using FFF have voids throughout the sample, while APF’s samples have voids located in between the contours. With two contours being printed on each layer, the interlayer gap between these contours cannot be filled effectively. This interlayer gap between contours was also present in the FFF samples; however, the voids are not just concentrated on this area.

### 3.3. Charpy Impact Testing

**Figure 4:**
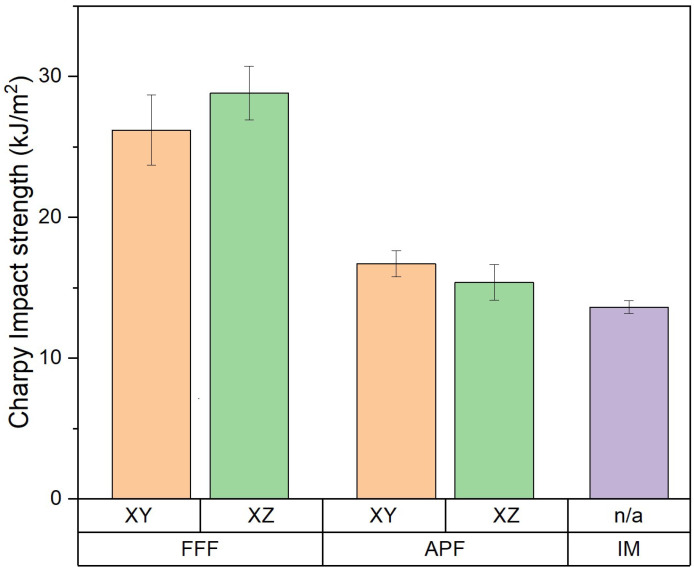
Charpy impact resistance of FFF-, APF-, and IM-manufactured samples (*n* = 10).

The impact resistance of the samples manufactured using APF, FFF, and IM was also assessed using a notched impact test (Figure 4). Samples manufactured using the FFF process were observed to have significantly higher impact strength than those of the other processes. FFF samples in the XY orientation (26.19 kJ/m^2^) and the XZ orientation (28.82 kJ/m^2^) showed a significant difference (*p* = 0.006). Samples manufactured using APF were assessed in both the XY orientation (16.71 kJ/m^2^) and the XZ orientation (15.39 kJ/m^2^), with no significant difference recorded (*p* = 0.694). APF samples in the XY orientation and IM samples (13.63 kJ/m^2^) showed a significant difference (*p* = 0.001).

### 3.4. Tensile Properties

**Figure 5:**
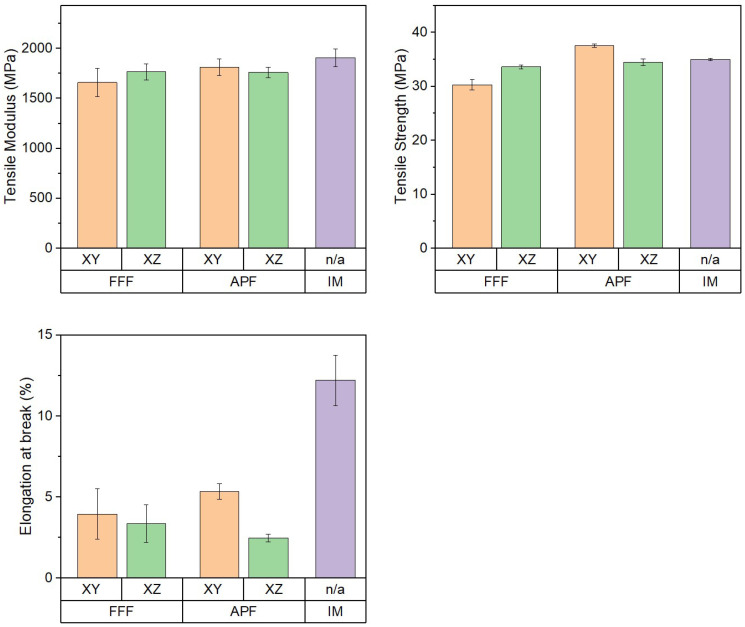
Tensile properties of FFF-, APF-, and IM-manufactured samples (*n* = 10).

The Young’s modulus, tensile strength, and elongation at break of the samples manufactured using APF, FFF, and IM were assessed using a tensile tester (Figure 5). The Young’s modulus of the samples manufactured using IM (1906.32 MPa) and APF in the XY orientation (1812.71 MPa) was assessed, with no significant difference recorded (*p* = 0.340). There was no significant difference between the FFF samples in the XZ orientation (1767.24 MPa) and the APF samples in the XZ orientation (1760.76 MPa) (*p* = 1.000). There was a significant difference between the APF samples in the XY orientation and the FFF samples in the XY orientation (1660.69 MPa) (*p* = 0.009).

The tensile strength of the samples manufactured using APF was assessed in the XY orientation (37.56 MPa) and the XZ orientation (34.48 MPa), with a significant difference recorded (*p* < 0.001). While the samples manufactured using APF in the XY orientation showed significantly higher tensile strength than those using the other processes/orientations, samples manufactured using APF in the XZ orientation and IM (34.99 MPa) were assessed, with no significant difference recorded (*p* = 0.522). The FFF samples in the XY orientation (30.32 MPa) achieved significantly lower tensile strength than those of the FFF samples in the XZ orientation (33.62 MPa) (*p* < 0.001).

The elongation at break of the samples manufactured using APF was assessed in both the XY orientation (5.42%) and the XZ orientation (2.71%), with a significant difference recorded (*p* < 0.001). The IM samples (12.36%) were significantly higher than all AM samples. The samples manufactured using APF in the XY orientation showed a significant difference to those of the FFF-manufactured samples in the XZ orientation (3.52%) (*p* = 0.008); however, there was no significant difference in elongation at break between APF in the XY orientation and FFF in the XY orientation (*p* = 0.203).

### 3.5. Flexural Properties

**Figure 6:**
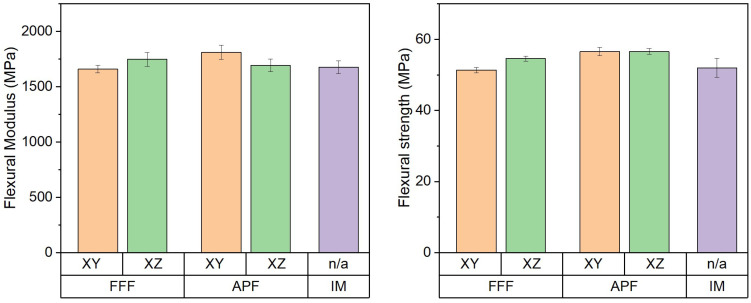
Flexural properties of FFF-, APF-, and IM-manufactured samples (*n* = 10).

The flexural modulus and flexural strength of the samples manufactured using APF, FFF, and IM were assessed using a three-point bending system (Figure 6). The flexural modulus of the samples manufactured using APF were assessed in both the XY orientation (1811.29 MPa) and the XY orientation (1692.74 MPa), with a significant difference recorded (*p* < 0.001). The samples manufactured using APF in the XY orientation showed a significant difference compared to the IM samples (1677.55 MPa) (*p* < 0.001). For the FFF samples manufactured in the XY orientation (1659.42 MPa) and the XZ orientation (1748.48 MPa), a significant difference was observed (*p* = 0.009). For both AM processes, there was a significant difference observed in the XY orientation (*p* < 0.001). The flexural strength of the samples manufactured using APF was assessed in both the XY orientation (56.56 MPa) and the XZ orientation (56.59 MPa), with no significant difference recorded (*p* = 1.000). For the FFF samples manufactured in the XY orientation (51.39 MPa) and the XZ orientation (54.57 MPa), a significant difference was observed (*p* < 0.001). Samples manufactured using IM (51.99 MPa) showed a significant difference to all AM samples except those manufactured using FFF in the XY orientation (*p* = 1.000). For the AM samples, the APF samples were observed to have significantly higher flexural strength than those of the FFF process.

### 3.6. Differential Scanning Calorimetry (DSC)

**Figure 7:**
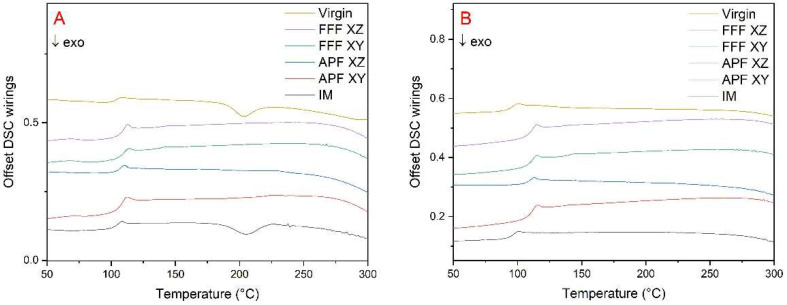
DSC first heating (**A**) and second heating (**B**) of virgin filament—IM-, APF-, and FFF-manufactured samples (*n* = 2).

The midpoints of the glass transition temperatures (Tg) of the samples were assessed using a DSC (Figure 7). Both the virgin (103.6 °C) and IM (103.4 °C) samples achieved comparable results. For the FFF-manufactured samples, the XY orientation had the highest midpoint temperature (109.3 °C), with the XZ having the lowest of the AM-produced samples (106.4 °C). For the APF samples, the XZ orientation (107.9 °C) temperature was slightly higher than that of the XY orientation (106.6 °C). For both the virgin and IM samples, an exothermic event occurred between 190 °C and 215 °C during the first heating cycle. This event did not occur in the second heating (Figure 7B), which would suggest that the event was process-related.

### 3.7. Dynamic Mechanical Thermal Analysis (DMA)

#### 3.7.1. Storage Modulus

**Figure 8:**
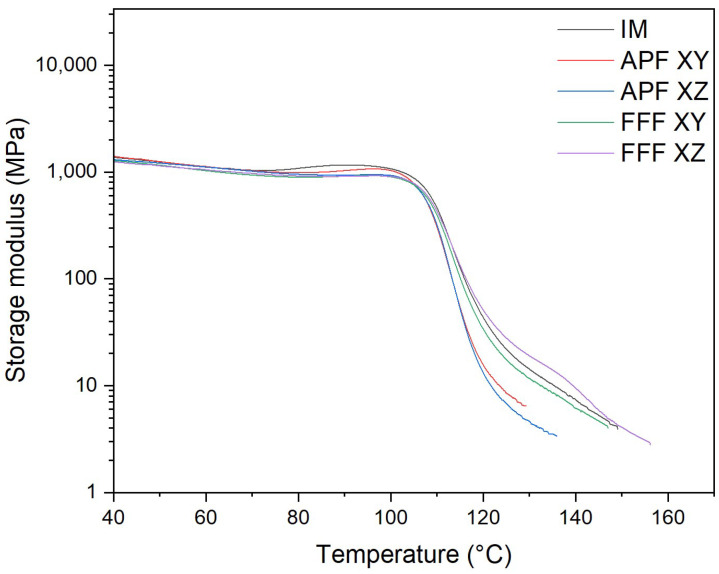
Storage modulus plot of APF-, FFF-, and IM-manufactured samples (*n* = 5).

The storage, loss modulus, tan delta peak, and Tg of the samples were assessed using a DMA. For the APF samples, the XY orientation (1373.6 MPa) had a significantly higher storage modulus (Figure 8) than the XZ orientation (1165.2 MPa) (*p* = 0.005). APF in the XY orientation had a significantly higher storage modulus than the FFF sample in the XY orientation (1144.86 MPa) (*p* = 0.002). Both the APF samples in the XY orientation and the FFF samples in the XZ orientation (1268.8 MPa) did not have a significantly different storage modulus to those of IM (1379.8 MPa).

#### 3.7.2. Loss Modulus

**Figure 9:**
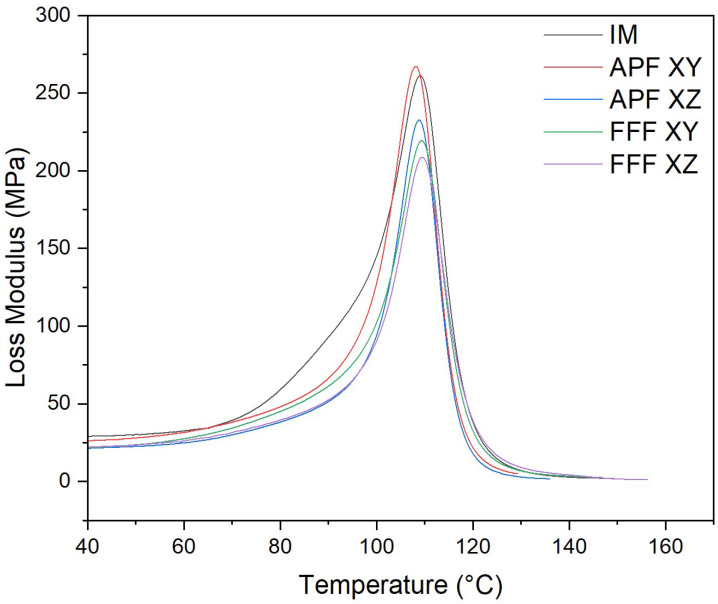
Loss modulus plot of APF-, FFF-, and IM-manufactured samples (*n* = 5).

Samples manufactured using IM (267.06 MPa) obtained the highest loss modulus (Figure 9), but it was not significantly different from the APF samples in the XY orientation (256.44 MPa) (*p* = 1.000). For the APF samples, the XY orientation was significantly higher than the XZ orientation (210.36 MPa) (*p* < 0.001). For the samples manufactured in the XY orientation, the APF samples were significantly higher than the FFF samples (205.12 MPa) (*p* = 0.001). For the FFF samples, there was not a significant difference between the XY orientation and the XZ orientation (212.06 MPa) (*p* = 1.000).

#### 3.7.3. Tan δ

**Figure 10:**
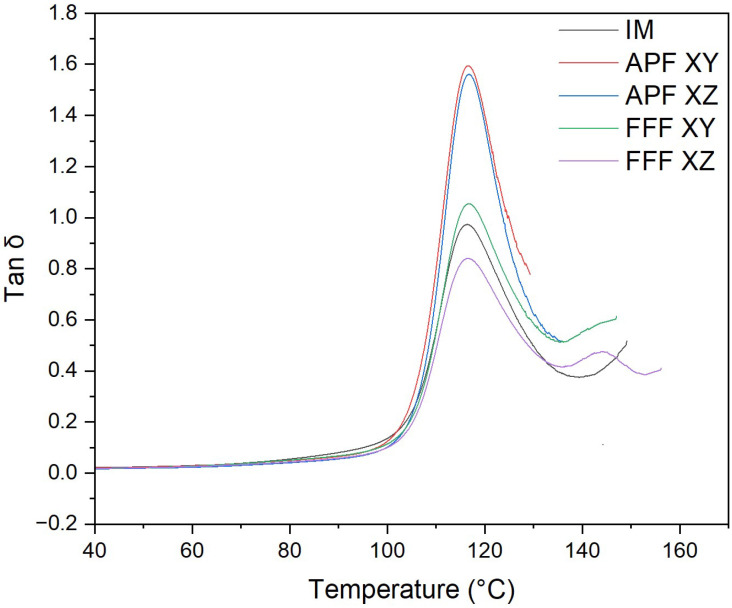
Tan delta plot of APF-, FFF-, and IM-manufactured samples (*n* = 5).

Samples manufactured using APF obtained the highest tan delta peak (Figure 10), with the XY orientation (1.4948) and the XZ orientation (1.4982) not significantly different (*p* = 1.000). The lowest tan delta peak was achieved using FFF in the XZ orientation (0.83082), which was significantly lower than every sample, including the FFF sample in the XY orientation (1.045) (*p* < 0.001). The sample manufactured using FFF in the XY orientation was not significantly different than the IM samples (0.96448) (*p* = 0.118).

#### 3.7.4. Glass Transition Temperature

The temperature at the tan delta peak was chosen as the Tg of the samples. For the APF samples, there was not a significant difference between the Tg of the XZ orientation (117.3 °C) and that of the XY orientation (116.97 °C) (*p* = 1.000). For the FFF samples, the Tg of the XY orientation (116.9 °C) and the XZ orientation (116.54 °C) was not significantly different (*p* = 1.000). The Tg of the AM samples were not significantly different from each other; however, the IM samples (115.29 °C) were significantly lower than any other samples.

### 3.8. Melt Flow Index (MFI)

**Figure 11:**
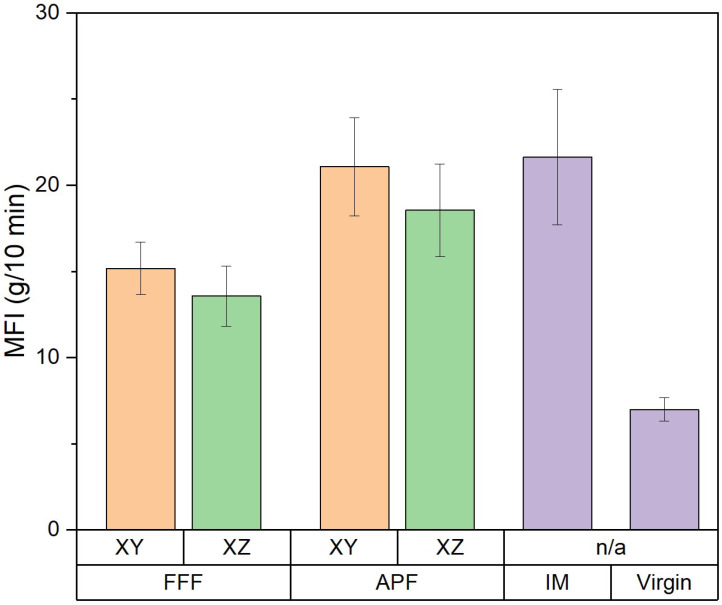
Melt flow index comparison for the three processes (*n* = 5).

The Melt Flow Index (MFI) of the samples manufactured using APF, FFF, and IM was assessed and compared to the virgin filament used in this study, as shown in Figure 11. The MFI of the IM samples (21.628 g/10 min was the highest among the samples but was not significantly higher than the APF samples in the XY orientation (21.072 g/10 min) (*p* = 1.000) or the APF samples in the XZ orientation (18.556 g/10 min) (*p* = 0.908). The APF samples in the XY orientation had a significantly higher MFI than the FFF samples in the XY orientation (15.180 g/10 min) (*p* = 0.014) and the FFF samples in the XZ orientation (13.564 g/10 min) (*p* = 0.001). The APF samples in the XZ orientation did not have a significantly higher MFI compared to the FFF process in either the XY (*p* = 0.609) or the XZ (*p* = 0.058) orientations. All processes had a significantly higher MFI compared to the virgin filament (6.984 g/10 min).

### 3.9. Capillary Rheology

**Figure 12:**
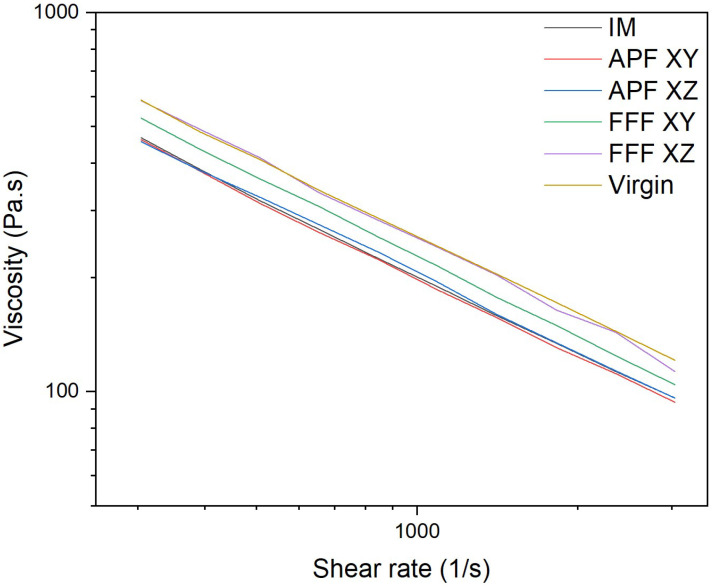
Viscosity vs. shear rate comparison for the three processes (*n* = 3).

The viscosity at varying shear rates of the samples manufactured using APF, FFF, and IM were assessed and compared to the virgin filament used in this study, as shown in Figure 12. For this comparison, a shear rate of 1411.2 1/s was chosen. There was no significant difference between the virgin filament and the samples manufactured using the FFF process in the XY orientation (*p* = 0.177) and the XZ orientation (*p* = 1.000). The samples manufactured using the FFF process in the XY orientation were not significantly different from IM (*p* = 1.000) or from APF in the XY (*p* = 1.000) and XZ orientations (*p* = 0.596). The IM samples were not significantly different from those of APF in the XY (*p* = 1.000) and XZ orientations (*p* = 1.000). There was a significant reduction in viscosity from the virgin filament to IM (*p* = 0.016) and APF in the XY (*p* = 0.006) and XZ orientations (*p* = 0.003). There was a significant difference between FFF samples manufactured in the XZ orientation and samples manufactured using IM (*p* = 0.011) and using APF in the XY (*p* = 0.004) and XZ orientations (*p* = 0.002).

## 4. Discussion

It is evident from the results that part density has a significant influence on the mechanical properties of the manufactured samples. For the AM samples, the inclusion of porosity acts as a stress absorber when under impact stresses. This stress absorption can reduce strain in the surrounding material by distributing the stress throughout the sample. FFF’s inherent limitations can prevent it from achieving 100% dense parts. The process of printing layer-by-layer can cause a distinct boundary between each layer, increasing porosity. As the number of layers increases, the number of voids also increases, resulting in a higher impact strength in XZ in FFF printing. The interlayer voids of the FFF parts were observed in the SEM images, with a high number of voids throughout the sample. The fracture behaviour of the FFF samples is similar to the findings of Mishra et al. [1]. The fracture followed the infill of the sample with a continuous crack at 45 degrees starting from the notch. The crack of the impact sample follows that of the interlayer voids shown in the SEM images. This stress absorption reduced the strain on the material, causing an increase in the samples’ impact strength. Mishra et al. [1] compared the impact strength of 3D-printing samples with varying infill patterns and infill percentages and found that the line pattern achieved the best impact strength. It was also stated that impact strength depends on the sample’s internal porosity and pattern, with the impact strength increasing as the infill density decreases until it reaches an 85% infill density. After this density, the impact strength begins to reduce. The APF process produces higher-density samples than the FFF process by dynamically adjusting material pressure in real time to control droplet size while maintaining a consistent DAR. This allows APF to fill in voids left from the previous layer, as areas with a lower height will require a larger droplet to maintain the same part height. By alternating the printing angle by 90 degrees, the droplet’s path should align with the previous layer’s trough, filling in the voids and creating better inter-layer adhesion. This increase in density reduces the impact resistance of the APF samples.

Another plausible reason for the reduction of impact strength could be the shear degradation occurring in the barrel of the APF and IM machines [20,26]. The FFF process does not apply much shear stress to the material, so filament manufactured for FFF would see a reduction in molecular weight when applied to higher shear stresses than those of the IM machine. Wang et al. [27] compared the impact strength of 3D-printed and IM PLA samples and found that the degradation of molecular weight was larger in the IM samples due to the high heat and pressure exerted on the samples during manufacturing. They also found that samples manufactured with higher platform temperatures saw an increase in crystallinity compared to IM, with samples manufactured with a 0.2 mm layer height and a 160 °C platform temperature achieving 114% of the impact strength of IM. This was a similar result to that of the FFF samples in the XZ orientation, which achieved a 112% increase in impact strength, as seen in Figure 4. The lower mould temperature of the IM samples compared to the APF and FFF build chambers and platform temperatures, respectively, could have allowed the 3D-printed samples to crystallize more, which would increase the impact strength of these samples. From the DSC results obtained, there is no perceived crystallization occurring in any AM process. For both the virgin material and IM samples, an exothermic event occurred between approximately 190 °C and 215 °C. This could be caused by rapid cooling associated with filament manufacturing and IM processing. The rapid cooling of the amorphous polymer may have caused frozen-in residual stresses to occur, which would make the samples more brittle. While the IM process rapidly cools the part, the AM process allows the part to cool down slowly as the layers are being printed layer-by-layer, reducing the residual internal stresses acting on the part. This reduction in internal stress could be seen when warping occurred in the initial AM parts. A brim around the part was used to increase build-plate adhesion and reduce this warping. This was done for all orientations and AM processes.

The thermo-mechanical characterization of the PC/ABS filament was assessed using DMA. The storage modulus (E’) is the measure of the energy stored elastically during deformation, while the loss modulus is the measure of energy converted to heat [28]. The samples manufactured via the IM process achieved the highest storage and loss modulus of all samples, which would indicate a good adhesion between the PC and ABS matrices [29]. Both the samples manufactured using FFF and APF in the XY orientation have a storage modulus not significantly lower than the IM samples, which would indicate good polymer matrix adhesion. However, both AM processes in the XZ orientation were significantly lower than the IM samples. This lower polymer matrix adhesion could be attributed to the increased layers that occur when printing in the XZ orientation. Like the storage modulus, samples manufactured via the IM process achieved the highest loss modulus, which was not significantly higher than that of samples manufactured in the XY orientation using APF. A higher loss modulus indicates an increase in frictional interactions between the polymer matrices, which enhances the dissipation of energy [30]. Both FFF samples and APF samples in the XZ orientation achieved a significantly lower loss modulus than samples manufactured using IM and APF in the XY orientation. A lower tan delta peak indicates an improvement in the viscoelastic properties of the PC/ABS filament. Samples manufactured in the XZ orientation achieved the lowest tan delta, which was significantly lower than any other sample. The APF samples achieved the highest tan delta, which indicates poorer viscoelastic properties compared to the IM and FFF processes. This could be due to the lower temperature needed to print the samples, as, unlike the FFF process, the material is deposited at high pressure. For the AM process, there was not a significant difference between the Tg of the samples. However, there was a significant reduction in Tg for samples manufactured using the IM process. This could indicate a reduction in molecular weight caused by an increase in shear stress. As seen from the MFI results, there is a significant increase in MFI for each process from the virgin filament. This would indicate that there is a reduction in molecular weight in the processed samples. There was not a significant difference between the IM and the APF samples, which would suggest that the processes degrade the material at similar rates. The IM samples and the APF samples in the XY orientation had a significantly higher MFI than the FFF samples, which would indicate that the FFF process had less impact on the molecular weight of the samples. Similar behaviour was found by Bassand et al. [20] and Wang et al. [27], where the APF and IM samples had lower molecular weight than the FFF process samples. For those tests, only the XY orientation was compared, whereas this study showed that the MFI of the APF samples in the XZ orientation did not exhibit a significant difference from the FFF samples in either direction, which would indicate that there was not a significant difference in the molecular weight of these samples.

Similarly to MFI, there was a significant reduction in the viscosity of the IM and APF samples in both directions compared to the virgin filament, suggesting that the higher level of shear in these processes is causing a reduction in molecular weight, which lowers the viscosity of the polymer. The samples manufactured using the FFF process in both directions were not significantly different from the virgin filament, which indicates that there is less of a molecular weight drop caused by this process. FFF in the XY process obtained a viscosity that was not significantly higher than the IM and the APF samples, suggesting that there are higher levels of shear at this orientation. However, the biggest drop off in viscosity was obtained by the IM and APF processes, suggesting that the shear degradation using these processes is lowering the molecular weight, as Bassand et al. [20] and Wang et al. [27] suggest.

Similarly to impact strength, flexural strength depends on the molecular weight and density of the polymer. IM causes more shear forces to act on the polymer, causing a greater degree of degradation to occur when compared to FFF printing [27]. This reduction in flexural properties in the IM samples could be attributed to the molecular weight change occurring in this process. While APF achieved the highest flexural strength of the three machines, the molecular weight changes are also higher in APF compared to the FFF process [20]. The IM flexural properties could have been influenced by the crystalline regions caused by rapid cooling. This increase in residual stress caused by the IM process could have contributed to its lower flexural properties. The porosity in the FFF samples could have acted as stress concentrations when subjected to a flexural load. This would have caused a reduction in the flexural strength of the FFF samples.

For the FFF process, the lower tensile properties could be due to the filling pattern or lack of active build-chamber heating, as the mechanical strength of these parts is influenced by the heat transfer between rasters [4]. The raster angle of the line pattern was ±45°, which is the same pattern used on APF. For the FFF process, a raster angle of 0/90 would manufacture samples with the highest tensile strength [31]. The lack of active heating in the FFF process would have caused weaker layer adhesion and could cause delamination and result in lower tensile properties. This delamination was observed in the SEM images of the XY FFF printed samples, which could be attributed to the lower tensile strength of these samples. Hecker et al. [10] found that FDM-printed samples obtained a significantly higher tensile strength and tensile modulus in the XZ orientation than in the XY. The pattern for the tensile modulus and tensile strength matches that of the impact strength, with the XZ orientation having higher properties in the FFF process and the XY orientation having higher properties in the APF process. Hentschel et al. [17] obtained a similar relationship with PMMA samples manufactured using APF. Samples manufactured in the XY orientation have higher tensile properties than those of the XZ orientation.

APF’s results were more similar to IM than to the FFF process. The plasticizing barrel for both IM and APF could have caused shear degradation to occur on the FFF filament, which could have lowered the material properties [20,26]. To get a fair comparison between APF and IM, an IM-grade material could be used, which would be able to withstand the shear forces during plasticizing without much molecular weight change. However, using an IM-grade material for the FFF process would see a variation in density and mechanical properties, as the material might not reach the viscosity requirements needed for this process. This was observed by Pinter et al. [9], who used an IM grade of ABS and found that the FFF process exhibited inconsistent density due to the variation in material flow characteristics caused by the change in diameter of the filament.

## 5. Conclusions

In conclusion, this study compared the mechanical and thermal properties of PC/ABS components manufactured via APF, FFF, and IM. Each manufacturing process exhibited unique characteristics, strengths, and weaknesses. While the FFF samples had a superior impact strength compared to the other manufacturing processes, the porosity, which acted like a stress absorber under an impact load, acted like a stress concentrator during a flexural load, resulting in a lower flexural strength. Compared to APF and IM, the FFF process obtained the worst tensile properties, which were caused by inadequate layer adhesion. Achieving only half the impact strength of the FFF process, shear degradation caused by increased heat and pressure inside a plasticizing barrel for both the APF and IM processes would have caused a reduction in the mechanical properties of the samples. The rapid cooling of the polymer during the IM process could have caused a reduction in mechanical properties due to an increase in residual stress. APF’s mechanical properties were comparable to those of IM, with the process achieving the highest tensile strength, highlighting its potential for producing robust PC/ABS samples. This research contributes valuable insights into the mechanical properties of PC/ABS samples manufactured via FFF, APF, and IM.

## Data Availability

The original contributions presented in the study are included in the article, further inquiries can be directed to the corresponding author.
